# Supplementary material for: Activity-induced Ca2+ signaling in perisynaptic Schwann cells of the early postnatal mouse is mediated by P2Y1 receptors and regulates muscle fatigue
Source: eLife. 2018 Jan 31;7:e30839. doi: 10.7554/eLife.30839 (PMC5798932; doi:10.7554/eLife.30839)
Supplement: Source code 1. — Each method is broken down conceptually into several categories, including variables needed, storage buffers, and approach taken. This logic can be utilized across a wide variety of software programs. [file elife-30839-code1.docx]

**Creation of Standard Deviation (SD) Maps (Ca^2+^ signals)**

**Info**

Standard deviation maps are similar to amplitude maps (max-min) but provide more detail of background noise and signal (Ca^2+^ event) to noise. There are 2 main ways to calculate SD maps: 1) over the entire stack (including before and after stimulus) or 2) separate calculations for before and after the stimulus. The first (entire stack) method is the simplest and displays active Ca^2+^ transients as an increased SD. The second method requires additional calculations but can standardize Ca^2+^ transient amplitude in units of decibels (dB). Note there may be more efficient ways to calculate standard deviation from image. Temporary buffers were created to provide a visual feedback to ensure the calculations were correct.

**METHOD 1: OVERALL (ENTIRE STACK) SD**

**Variables needed:**

- size of stack (width, height)

- depth of stack (number of slices)

**Storage buffers:**

SUM_FRAME (unsigned long). size = width * height;

AVG_FRAME (float or double). size = width * height;

SD_FRAME (float or double). size = width * height;

**Approach:**

• Loop 1 (from top of stack to bottom of stack)

- extract intensity value from every XY pixel in each slice

- add the pixel intensity to existing values in the SUM_FRAME

• Create the AVG_FRAME by dividing the every XY pixel in the SUM_FRAME by the number of slices

• Loop 2 (from top of stack to bottom of stack)

- extract intensity value from every XY pixel in each slice

- subtract the intensity value from the corresponding XY pixel in the AVG_FRAME

- square the result and write the squared result into the SD_FRAME at the same XY pixel location.

**Finalize:**

• For every XY pixel in the SD_FRAME, divide the value by number of slices in the stack -1, and take the square root of that value. This is called an SD Map.

-i.e. sqrt( (SD_FRAME_XYPIXVAL / (number of slices -1) );

**METHOD 1a: Pre-stimulation versus stim (Imaging dB)**

**Additional storage buffers:**

MAX_FRAME (integer). size = width * height (zero all the values in the MAX_FRAME);

MIN_FRAME (integer). size = width * height (set all the values in the MIN_FRAME to max (e.g. 65535));

dB_FRAME (float). size = width * height

**Approach:**

• Loop 1 (use only those slices before stimulation)

- Create an SD map from the pre-stimulation period using Method 1 (above)

• Loop 2 (use only those slice during/after stimulation)

- compare XYPixel in slice to XYPixel in MAX_FRAME. If greater than max frame, write value into the max frame

- compare XYPixel in slice to XYPixel in MIN_FRAME. If less than min frame, write value into the min frame

**Finalize:**

• Using the Pre-stim SD frame, max & min frames, create a map of imaging dB (dB_FRAME) by using the following formula. For every XY pixel:

- Imaging dB = log10 ((Max Map XYpixel - Min Map XYpixel) / SD Map XYpixel) x 20

- i.e.

- Calculate Max-Min to get intensity amplitude. Take the Log10 of this value

- Extract the pre-stim SD value.

- divide the log10 amplitude value by the pre-stim SD value, then multiple the result by 20

***note - you may want to apply a scaling factor to the values to stretch if over more of the LUT.

***note - a shortcut is to use SD of the intensity change during stimulation instead of MAX-MIN, but you would need to specify the Imaging dB as log10((SD during / SD before))x20

**METHOD 2: Spatio-tempoal (ST) Map create (using bitmasks)**

**Variables needed:**

- size of stack (width, height)

- depth of stack (number of slices)

**Storage buffers:**

MASK_FRAME (boolean). size = width * height;

STMap (unsigned short). size = width (or height) * number of slices (depth)

DDT_STACK (ubyte, unsigned short). size = width * height * number of slices (depth)

Intensity_Sum (integer)

**Approach (Masking):**

- to display only those actively fluorescing cells, take the differential of the stack.

- find the approximate rise time of the Ca2+ transient (in frames) divide by 2 and round up to the nearest odd number. (delta T wing size - with delta T = (wing size * 2) + 1.

Loop 1 (from delta T wing size to (depth - delta T wing size) in the original movie)

- for every XY pixel in the current frame, calculate (current frame + delta T wing size) - (current frame - delta T wing size)

- write this value into the corresponding slice in the DDT Stack.

then…

- calculate the SD from the differential stack (DDT_STACK) using Method 1 above

- apply a Gaussian blur to the image (3x3 kernel, sd = 1.0-1.5)

- threshold the stack. Typical thresholding values ~ 12-14dB, or when background noise clumps ~ 5-9 pixels.

- switch the thresholded pixels to TRUE (1) in the MASK_FRAME

**Approach (ST Map):**

- dispose of the DDT_STACK

Loop 1 (from start to finish slice in the original stack)

- Determine the axis of averaging (horizontal or vertical)

- For vertical averaging:

- for every X pixel across the image

- reset the Mask_Count variable and Intensity_Sum

- check every Y pixel at that X position.

- if the XY pixel is TRUE (1) in the MASK_FRAME, add the intensity value to the Intensity_Sum variable

- at the end of the Y pixel column, divide the Intensity_Sum by the Mask_Count

- write this value at the X position in the vertical row of the ST Map corresponding to the slice number of the original stack.

- For horizontal averaging, step through the Y positions and average in the X direction

**Finalize:**

• Calibrate and adjust the brightness/contrast levels to stretch the averaged intensity value range over the full LUT.

**METHOD 3: Tissue distortion using edges (for muscle shortening)**

**Variables needed:**

- size of stack (width, height)

- depth of stack (number of slices)

**Storage buffers:**

as for Method 2 (above)

left_position (integer)

right_position (integer)

distortion_array (float) size = number of slices in stack

**Approach:**

- determine the direction of distortion

- create and ROI over the tissue ensuring that it extends beyond the tissue edges

- create an ST Map (method 2 above) without masking ensuring that the averaging occurs parallel to the edges

- threshold the ST Map so that the edges are distinct from the background (or vice versa)

Loop 1 (from top to bottom of ST Map)

- for every row in the ST Map

- reset the left_position and right_position variables

- moving from left to right, store the position of the first thresholded pixel encountered in the left_position variable

- moving from right to left, store the position of the first thresholded pixel encountered in the right_position variable

- write the difference between the right and left position into the distortion_array.

**Finalize:**

• Calibrate and export as a trace.
